# Magnetic resonance tractography exhibiting retrograde degeneration of the corticospinal tract in a patient with a unilateral spinal cord tumor

**DOI:** 10.1002/brb3.2020

**Published:** 2021-02-27

**Authors:** Yusuke Osaki, Wataru Sako, Masafumi Harada, Yuishin Izumi

**Affiliations:** ^1^ Department of Neurology Tokushima University Hospital Tokushima Japan; ^2^ Department of Radiology Tokushima University Hospital Tokushima Japan

**Keywords:** corticospinal tracts, diffusion tractography, retrograde degeneration, spinal cord neoplasms

## Abstract

**Background:**

Transection‐induced axonal retrograde degeneration, in contrast to Wallerian degeneration, has not been widely recognized in clinical practice.

**Aims of the Study:**

To assess a potential of corticospinal tractography for detecting axonal retrograde degeneration.

**Methods:**

We assessed the corticospinal tractography of a 74‐year‐old woman with monoplegia of the lower limb due to a unilateral thoracic spinal cord tumor.

**Results:**

The tractography revealed integrity reduction of the corticospinal tract in the cerebra contralateral to the spinal cord tumor.

**Conclusions:**

The present report supports that magnetic resonance tractography has the potential for detecting this under‐recognized phenomenon.

## Graphical abstract

To assess a potential of corticospinal tractography for detecting axonal retrograde degeneration, an under‐recognized phenomenon, we performed the cerebral corticospinal tractography of a 74‐year‐old woman with monoplegia of the lower limb due to a unilateral thoracic spinal cord tumor. The tractography revealed integrity reduction of the corticospinal tract in the cerebra contralateral to the spinal cord tumor. The present report supports that magnetic resonance tractography has the potential for detecting this under‐recognized phenomenon.

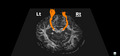

## 1 INTRODUCTION

Recent studies have shown that axon degeneration precedes, and sometimes causes, neuronal death in several disorders: motor neuron disease, Alzheimer's disease, Parkinson's disease, Huntington's disease, multiple sclerosis, hereditary spastic paraplegia, peripheral neuropathies, and glaucoma (Coleman, 2005; Conforti et al., 2014) Local disconnection of an axon causes degeneration of the remaining part. The degeneration occurs from the cut site both toward the distal axonal terminal and toward the proximal cell bodies (Sanes et al., 2013) While distal axonal degeneration is well known as Wallerian degeneration and has attracted much attention, proximal retrograde degeneration has not been well reported, except for animal studies or autopsy studies in humans (Coleman, 2005; Fishman, 1987; Kanamori et al., 2012; Knöferle et al., 2010; Vincent et al., 2006; Yamamoto et al., 1989) Retrograde degeneration should be considered as a factor of poor prognosis in various neuropathological conditions, especially spinal cord injury (Koskinen et al., 2014; Seif et al., 2019) Estimating the degree of the retrograde degeneration can provide reliable criteria, or a quantitative approach for the efficacy of clinical trials, as is already considered in spinal cord injury (Seif et al., 2019).

Magnetic resonance imaging (MRI) is an approach to recognize the retrograde degeneration derived from spinal cord injury. Routine MRI cannot specifically visualize subtle axon pathology in the central nervous system (CNS). However, diffusion tensor imaging (DTI) can clarify the geometric aspect of white matter in the CNS, which can help us to discern subtle axon pathology; hence, the technique is a reliable way for detecting and quantitating the secondary axonal degeneration in spinal cord injury (Seif et al., 2019).

Previous studies that adopted DTI to detect changes in the corticospinal tract (CST) targeted tetraplegic patients rather than patients with a unilateral spinal lesion (Freund et al., 2012; Koskinen et al., 2014; Wrigley et al., 2009) If a study were to target patients with a unilateral spinal lesion, the study could compare side to side and thus provide better internal control. We, therefore, observed a case of unilateral spinal cord compression by an extramedullary tumor. The case exhibited reduction of CST fibers in the contralateral cerebra by MR tractography with DTI. Our report supports that MR tractography has the potential to evaluate the retrograde degeneration of nerve tracts in the CNS.

## 2 CASE STUDY

A 74‐year‐old woman presented with an 8‐month history of left lower‐extremity weakness. In November 2016, she began to develop a gait disturbance, left lower‐extremity weakness, and hypoesthesia. In January 2017, she noted gradual onset of urinary urgency. Her gait disturbance progressed, and by June 2017, she was only able to walk with assistance by caregivers. In July 2017, she visited our department. On neurological examination, she showed no abnormalities of the cranial nerves or upper extremities. She exhibited moderate weakness, hyperreflexia, and pathological reflex in the left lower extremity. She also showed loss of vibration sense with left‐side dominance, whereas touch, pain, and temperature sensation were impaired symmetrically. The clinical findings suggested left dominant corticospinal and posterior column tract impairment.

Needle EMG in the left tibialis anterior and vastus medialis showed poor activation and normal recruitment. Spontaneous discharges were not observed. Nerve conduction study showed symmetrically and mildly decreased sensory nerve action potential of the sural nerve, whereas short‐latency tibial nerve somatosensory evoked potential showed poorly evoked and mildly prolonged waveform of the cortical P38 potential with left stimulation. Neurophysiological testing supported left dominant posterior column tract impairment.

**FIGURE 1:**
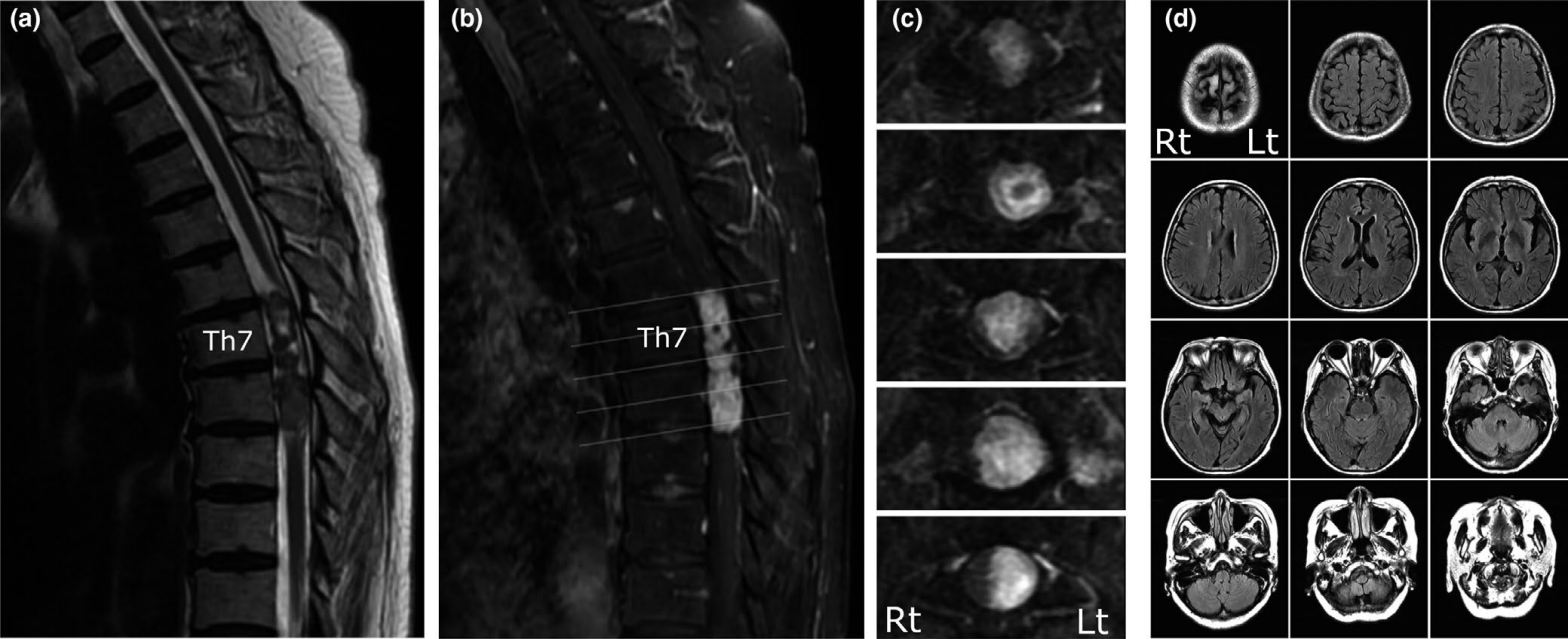
Spinal cord and brain MRI. MRI demonstrated the unilateral spinal cord tumor and retrograde regression of the corticospinal tract (CST) fibers. T2‐weighted images showed mixed intensity signal mass in the thoracic spinal canal at the T7 and T8 levels (a). T1‐weighted gadolinium‐enhanced images showed an enhanced intradural extramedullary mass which was located on the left side of the spinal cord and displaced the spinal cord to the right (b, c). Fluid‐attenuated inversion recovery images in the brain seemed normal (d)

Magnetic resonance imaging demonstrated the extramedullary spinal tumor and no comorbid lesions in the brain. T2‐weighted images showed a mixed intensity signal mass in the thoracic spinal canal at the T7 and T8 levels (Figure 1a). T1‐weighted gadolinium‐enhanced images showed an enhanced intradural extramedullary mass that was located on the left side of the spinal cord and displaced the spinal cord to the right (Figure 1b,c). Schwannoma was considered the most likely as indicated by the radiological feature. In order to rule out comorbid lesions, we performed routine MRI in the brain and the cervical spinal cord. These MRI, including fluid‐attenuated inversion recovery images in the brain, showed no abnormal lesions (Figure 1d).

**FIGURE 2:**
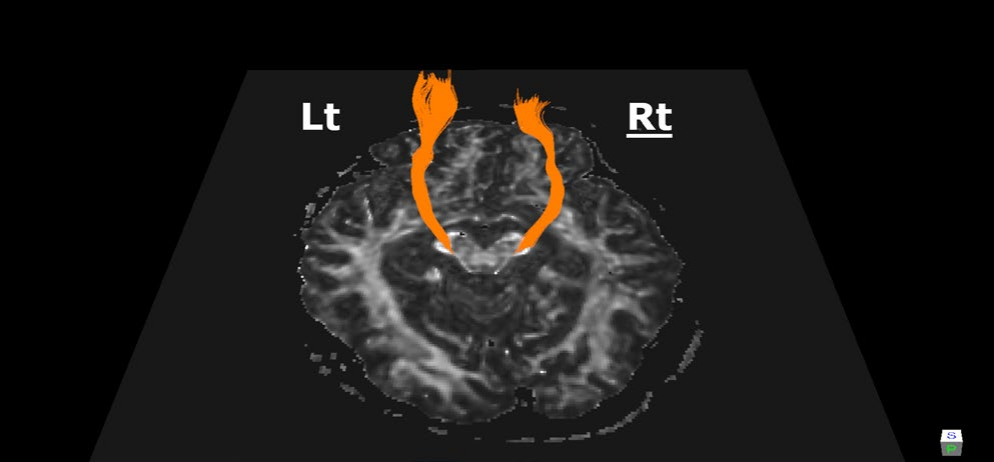
Corticospinal tractography. The tractography showed the reduction of the right CST fibers. Note that the right tract is shown on the right in the figure. The calculated track number was 232 on the right and 650 on the left (the cutoff for normal is 606). Also note that the CST seemed “truncated” on the right side

We attempted to quantitate the number of CST fibers in the cerebra with MR tractography on the assumption that retrograde degeneration of the CST fibers may occur from the spinal cord lesion. We obtained the patient's signature on the consent form approved from the Ethics Committee of Tokushima University Hospital. The DTI was acquired using a 3.0 T Discovery 750 scanner (GE Healthcare) with an 8‐channel head coil. We set scan parameters as follows: TR, 15,000 ms; TE, 84.9 ms; flip angle, 90; the field of view, 240 mm; matrix, 128 × 128; slice thickness, 2.5 mm; diffusion gradient directions, 33; and b value, 800 s/mm (Conforti et al., 2014). Briefly, eddy‐current distortion and head motion were corrected using the option of FSL (https://fsl.fmrib.ox.ac.uk/fsl/fslwiki/FSL). All fibers in the brain were reconstructed according to the fiber assignment by continuous tracking by TrackVis software (www.trackvis.org), and then, CST fibers were selectively visualized as the tracts between cerebral peduncle and precentral gyrus. The detailed method was described previously (Sako et al., 2015, 2019) The MR tractography revealed reduction of the right CST fiber density compared with the left (Figure 2). The track number, computed using the option of TracVis software, was 232 on the right and 650 on the left. The track number of the affected right side was decreased, compared with those of the normal controls reported in another previous study as 777 ± 57.5 (five males, five females; age, 68 ± 4.0 years) (Sako et al., 2019) Moreover, the right CST appeared “truncated” as observed in a previous report on cases of amyotrophic lateral sclerosis (Rajagopalan & Pioro, 2017) The mean fractional anisotropy value over the whole CST of this patient was 0.58 and 0.58 on each side, which was similar to those of the normal controls previously reported as 0.574 ± 0.007 (Sako et al., 2019).

The patient underwent surgical removal of the spinal cord tumor. The resected tumor was pathologically revealed as a plexiform schwannoma. The patient subsequently showed gradual improvement in her gait disturbance but did not achieve complete recovery. Her modified Rankin Scale, which was calculated as grade 3 at the first visit, remained the same.

## 3 DISCUSSION

The present case showed significant reduction of the supratentorial CST fibers using the deterministic tractography method. The fiber reduction was unilateral and on the contralateral side of an extramedullary spinal cord tumor. The patient had no other lesions in the central or peripheral nervous system; therefore, her reduction of CST fibers was surely derived from a retrograde effect due to the distal axonal damage.

The retrograde degeneration of the CST associated with spinal cord lesion has attracted the attention of clinicians. Fishman reported 12 autopsy cases with a history of spinal cord injury and rostrally expanded degeneration of the lateral columns (Fishman, 1987). Yamamoto et al. (1989) reported an autopsy case of hematomyelia at the cervical spinal cord and showed CST degeneration at the pontomedullary junction and disappearance of Betz large cells. These reports discussed about postmortem findings rather than findings concurrent with the injury. There have been also several antemortem reports demonstrating concurrent axonal degeneration in the brain due to spinal cord injury (Seif et al., 2019) Some of them adopted MR DTI methods, as in our study; however, they did not focus on the laterality of the lesions (Freund et al., 2012; Koskinen et al., 2014; Wrigley et al., 2009) To our knowledge, this case report is the first to show antemortem concurrent CST degeneration caused by a unilateral spinal cord lesion with an internal control.

Deterministic tractography allowed us to estimate and quantify the track number of supratentorial CST in the present case with a unilateral spinal cord compression. The quantification showed the track number along the CST of the affected side was reduced to one‐third of the unaffected side. On the other hand, the patient clinically showed moderate and irreversible motor dysfunction on the affected side. Consequently, the reduced track number possibly predicted her poor recovery of motor function. Quantification of the track number may be a predictor of outcome in spinal cord injury and various spinal cord diseases.

## CONFLICT OF INTEREST

None declared.

## AUTHOR CONTRIBUTION

YO contributed to design and conceptualization of the study, acquisition/analysis/interpretation of the data, and drafting/revising the manuscript. WS contributed to medical care for the patient, design of the study, acquisition/analysis/interpretation of the data, and drafting/revising the manuscript. YO and WS contributed equally to the manuscript. MH contributed to image acquisition and analysis/interpretation of the data. YI contributed to analysis/interpretation of the data and revising the manuscript.

### Peer Review

The peer review history for this article is available at https://publons.com/publon/10.1002/brb3.2020.

## Data Availability

The data that support the findings of this study are available on request from the corresponding author. The data are not publicly available due to privacy or ethical restrictions.
